# External costs of atmospheric Pb emissions: valuation of neurotoxic impacts due to inhalation

**DOI:** 10.1186/1476-069X-9-9

**Published:** 2010-02-19

**Authors:** Massimo Pizzol, Marianne Thomsen, Lise Marie Frohn, Mikael Skou Andersen

**Affiliations:** 1 Department of Policy Analysis, National Environmental Research Institute (NERI), Aarhus University, Denmark; 2 Department of Development and Planning, Aalborg University, Denmark; 3 Department of Atmospheric Environment, National Environmental Research Institute (NERI), Aarhus University, Denmark

## Abstract

**Background:**

The Impact Pathway Approach (IPA) is an innovative methodology to establish links between emissions, related impacts and monetary estimates. Only few attempts have so far been presented regarding emissions of metals; in this study the external costs of airborne lead (Pb) emissions are assessed using the IPA. Exposure to Pb is known to provoke impacts especially on children's cognition. As cognitive abilities (measured as IQ, intelligence quotient) are known to have implications for lifetime income, a pathway can be established leading from figures for Pb emissions to the implied loss in earnings, and on this basis damage costs per unit of Pb emission can be assessed.

**Methods:**

Different types of models are here linked. It is relatively straightforward to establish the relationship between Pb emissions and consequent increase in air-Pb concentration, by means of a Gaussian plume dispersion model (OML). The exposed population can then be modelled by linking the OML-output to population data nested in geo-referenced grid cells. Less straightforward is to establish the relationship between exposure to air-Pb concentrations and the resulting blood-Pb concentration. Here an Age-Dependent Biokinetic Model (ADBM) for Pb is applied. On basis of previous research which established links between increases in blood-Pb concentrations during childhood and resulting IQ-loss we arrive at our results.

**Results:**

External costs of Pb airborne emissions, even at low doses, in our site are in the range of 41-83 €/kg emitted Pb, depending on the considered meteorological year. This estimate applies only to the initial effects of air-Pb, as our study does not address the effects due to the Pb environmental-accumulation and to the subsequent Pb re-exposure. These are likely to be between one and two orders of magnitude higher.

**Conclusions:**

Biokinetic modelling is a novel tool not previously included when applying the IPA to explore impacts of Pb emissions and related external costs; it allows for more fine-tuned, age-dependent figures for the external costs from low-dose exposure. Valuation of additional health effects and impacts e.g. due to exposure via ingestion appear to be feasible when extending the insights from the present pilot study.

## Background

While environmental scientists tend to use physical units in order to measure and describe risks and impacts, the policy-making process is faced with trade-offs, that are often economic in nature. Attempts to express the environmental impacts of emissions in monetary terms have been presented in recent years [1-3]. Measures of the costs of environmental impacts are often controversial, but monetary estimates can be regarded as a novel indicator, that can inform and support the integration of environmental concerns into the policy-making process. In the context of micro-pollutants like toxic metals, figures for the external costs can be used to inform decision makers about the possible value of limiting these emissions, for instance from waste-to-energy (WtE) plants, which are currently increasing in significance in the energy sector as policy-makers are searching for more low-carbon fuels.

'Monetization' of the impacts of emissions is not always feasible, and is associated with a great deal of uncertainty. The Impact Pathway Approach (IPA) for assessment of the 'external costs' of air emissions, developed under the ExternE project series, has however gained recognition for reducing the biases associated with more pure, preference based willingness-to-pay estimates [2]. 'External costs' relating to air pollution arise from various emissions affecting society which are not factored in when prices are set. In this sense they are labelled 'external' (or 'externalities'). The IPA methodology was applied initially to classic environmental pollutants such as NO_x _and SO_2_, e.g. from energy sources such as power plants. More recently modern sources of emissions such as WtE plants, which emit certain persistent micro-pollutants (dioxins and metals), have been explored, but authoritative figures for the external costs are still lacking. The micro-pollutants represent a matter of concern due to their high toxicity and persistence in environmental media. WtE plants provide energy and heat from the combustion of municipal waste: they are often located in urban areas and the concentration of micro pollutants in their stack emissions depends on the municipal waste composition, which may be varying, and is therefore sometimes unpredictable [4]. For such reasons, WtE plants have faced requirements for expensive abatement measures. The estimates for the external costs can in this context be useful to inform Cost Benefit Analysis (CBA) as well as more comprehensive Life Cycle Assessment (LCA) of the waste management practices.

This study explores the possible external costs associated with atmospheric emissions of lead (Pb) from a WtE plant located in a suburb of Copenhagen, Denmark. Pb is a substance for which the negative impacts on human health have been established in numerous studies reported in the scientific literature [5-9], and for this reason the use of Pb has gradually been prohibited for more and more purposes. Even with these restrictions, the human exposure to Pb from different media (air, soil, water) is still significant, and the focus on a small source like the WtE plant is then justified; its contribution corresponds to 1% of the total Pb background exposure via inhalation (Pizzol M, Thomsen M, Andersen M.S.: Long-term human exposure to Pb from different media and intake pathways, submitted), but even small increments in Pb-exposure for the general population can provoke significant impacts. The aim of the study is to explore what monetary estimates one will arrive at by expanding the conventional impact pathway methodology with bio-kinetic modelling that can establish a precise relationship between concentrations of Pb in the air and resulting concentration of Pb in the blood. Because of our methodological interest the assessment is limited to the effects of Pb-air exposure: only the direct link between source and exposure via inhalation is considered, whereas the contribution from ingestion of Pb deposited to soil and after redistributed to water and biota is not included. Consequently only a part of the total costs related to Pb emissions from the studied source is here determined (approx. 4% according to Spadaro [1]). While the ingestion pathway contributes importantly to total daily intake of Pb, the uncertainties in linking the specific source studied here to increments in ingestion are significant. Still, the study provides a more firm basis for expanding the assessment to additional impacts, especially due to ingestion.

We take our point of departure in a site-specific study of Pb-emissions from a large WtE plant, but we argue that the findings have general validity beyond the specific site, provided that the specific circumstances of population density and the relative monetary values in Denmark are adjusted to other sites.

## Methods

**Figure 1:**
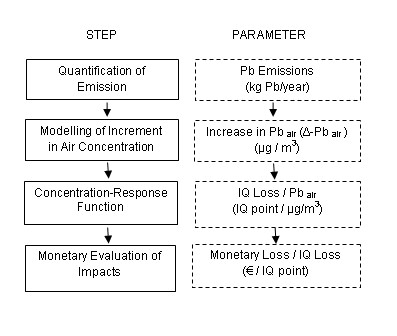
**Impact Pathway Approach**. Impact Pathway Approach (IPA): theoretical steps to the left side boxes from top to bottom. The output parameters from models used in each of the steps specified in the right side boxes from top to bottom, where Pb_air _quantifies the concentration of Pb in the air, Δ-Pb_air _quantifies the marginal increase in Pb_air _and IQ quantifies the Intelligence Quotient.

The IPA structure consists of four consecutive steps [2]: 1) Emission determination from the specific source; 2) Assessment of atmospheric dispersion of the contaminant in terms of increased concentration at receptor sites; 3) Choice of relevant dose-response functions quantifying specific impacts resulting from exposure to the contaminants in question; 4) Monetary evaluation of the selected impacts, and assessment of the external costs. The external costs of the emission can be calculated by multiplying the various parameters along the pathway. Steps in the IPA methodology and the corresponding parameters relating to the present case study on Pb are illustrated in Fig. 1.

### Exposure scenarios and modelling

#### Modelling of Pb dispersion

**Figure 2:**
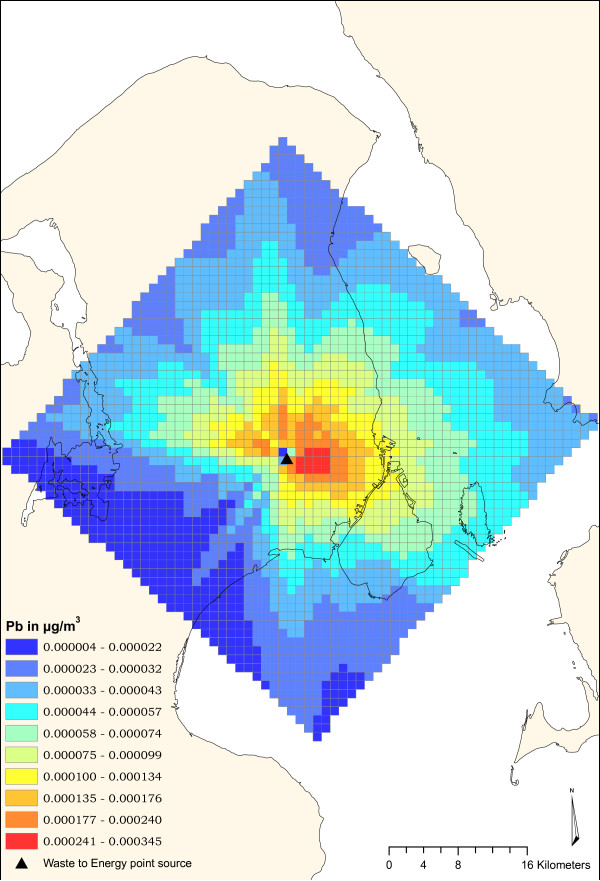
**Modelling of Pb dispersion**. Spatial distribution of calculated Δ-Pb_air _values around the point source (The WtE plant is represented by the black triangle in the middle). Values are in μg/m^3^.

This study analyses a WtE plant located in the suburbs of Copenhagen (Denmark). The plant considered in this study emitted 969 kg Pb in year 2000 in stack gases [10]. The Pb is mostly adsorbed to atmospheric particles (particulate matter - PM) which are suspended in the air [11-13]; the Pb concentration in the air is here defined as Pb_air_. The emission of Pb from the plant raises the existing level of Pb_air_. The concentration contribution from the source to the background level is defined as a 'delta concentration': Δ-Pb_air_. It is described as a "marginal" contribution, because the source is small compared to the sum of all the other existing sources contributing both to Pb_air _and to Pb exposure. The Danish Gaussian plume dispersion model (OML) [14,15] has been applied to model the Δ-Pb_air _values (Fig 2).

The domain of the model covers 50 × 50 km around the point source giving a total number of 1 × 1 km grid cells of approx. 2,500, all geo-referenced in UTM_32 _coordinates. A calculated Δ-Pb_air _value is assigned by the model to each grid cell in the model domain. Mean values of Δ-Pb_air _are 1 × 10^-5 ^μg/m^3 ^and the range comprises values of between 1 × 10^-4 ^and 1 × 10^-6 ^μg/m^3^. Values are calculated for three different years: 2000, 2001 and 2002 based on the same emissions but with different meteorological data for the three years. The concentration contribution from the point source (Δ-Pb_air_) is therefore, on average, two orders of magnitude lower than the background concentration of Pb_air_, as reported in Denmark's Air Quality Monitoring Programme [11] where the content of Pb in the PM_10 _(atmospheric particles with aerodynamic diameter less than 10 μm) was measured for traffic and urban background conditions. Values of Pb_air _= 0.0079 (μg/m^3^) for traffic and Pb_air _= 0.0039 μg/m^3 ^for urban background conditions respectively have been measured in the city of Copenhagen [11].

#### Processing of population data

Population data for Denmark have been distributed on the same grid cells as applied in the calculation of the Δ-Pb_air _data. The population data for the age distribution of the inhabitants in each grid cell have been retrieved from the Danish CPR-registry (Personal Registration Code -CPR). The population under exposure can be derived by crossing the two distributions (population and Δ-Pb_air _values). In this way, the grid cells where no additional Pb_air _occurs or no population is reported will not contribute to the calculation of external costs.

The damage window for the impact of Pb explored in this study is cognitive impairment in children resulting from Pb exposure. Neurodevelopment is critical during childhood, especially the first 2-3 years of life [1,16,17]. For this reason the relevant segment of the population for the present study is the number of children aged between 0 and up to 3 years in each cell. The total vulnerable population under exposure in this case study includes almost 60,000 children between the age of 0 and 3.

### Pb Concentration Response Function (CRF)

The Pb concentrated in the air is inhaled and then absorbed in the body, and increases the blood Pb concentration (Pb_blood_). Pb is known to provoke cognitive impairment in children; such impact is measured in terms of Intelligence Quotient (IQ) loss in children and related to increases in the Pb_blood _[16,18,19]. The Concentration Response Function (CRF) in equation 1 determines the relationship between an increase in Pb_air _and the corresponding increase in Pb impacts.

The CRF linking Pb_air _and the IQ loss: IQ_loss _- Pb_air _(IQ lost point/μg/m^3 ^Pb_air_) can then be calculated as the product of two functions (1):

1) Pb_blood _- Pb_air _(μg/dl Pb_blood_/μg/m^3 ^Pb_air_), the relationship between an increase in Pb_air _and the corresponding increase in Pb_blood_.

2) IQ_loss _- Pb_blood _(IQ lost point/μg/dl Pb_blood_), the relationship between an increase in Pb_blood_, and a loss of IQ points.(1)

Values and references for the two functions 1) and 2) are presented in detail in the following sections (cf. sect. 2.2.2. and 2.2.3.).

### Long-term exposure assessed with ADBM

Previous studies determined the Pb_blood _- Pb_air _function by means of statistical techniques assessing the relationship between measured media concentration and bio-monitoring values [20,21]. In this study it has been calculated by using the Age-Dependent Biokinetic Model (ADBM) [22] for Pb from the International Commission for Radiological Protection (ICRP), in order to account for human biokinetics and age-time conditions. The model has previously been extensively described and validated [22-24]. The model considers the human body as a system of more than 20 different interrelated compartments. The continuous exchange of Pb between compartments follows linear first-order kinetics and is described by different Pb-specific distribution rates, depending on the characteristics of each single compartment. Pb accumulates e.g. in bone, liver and brain, and these compartments work as reservoirs of Pb, releasing and redistributing the metal slowly as a function of time. The utility of the model is that it can determine the Pb_blood _after defined periods of exposure and for different age categories or physiological characteristics. In this specific case-study we only include the contribution from inhalation to the resulting concentration in blood. The amount of Pb that is daily absorbed in the body (Pb_absorbed_), expressed in μg/day, is the input to the ADBM model and has been estimated according to equation (2):(2)

Where IR_inhalation _is the breathing rate in m^3^/day, and AF_Pb _is the Absorption Factor for Pb via inhalation expressed as percent of Pb intake.

The breathing rates and Pb absorption rates are the key parameters determining the actual Pb absorption needed as input for the ADBM. Reference values for age-dependent breathing rates, representing the amount of air (m^3^) inhaled daily by children and adults have been adopted as reported by US EPA [25,26]. The absorption factor, AF, represents the fraction of Pb inhaled by air that enters the body. While breathing rates differ between children and adults, the absorbed fraction by inhalation is similar and is estimated to be 0.37 [22,27]. No thresholds for intake have been identified in the literature and we assume that there will be intake at the relevant levels of Pb_air _(cfr. sect.2.1.). The relation between Pb_air _and Pb intake is then, for the range of concentrations here considered, assumed to be linear for marginal increases in Pb_air_.

### Determination of the Pb air - Pb blood age-dependent function

Pb_blood _as output from the model is a function of the age, the duration (time) of exposure, and the Pb_air_. The model is nonlinear for constant values of Pb_air _(the increase of Pb_blood _in time is exponential and reaches an equilibrium value). When age/time exposure conditions are instead fixed, Pb_blood _is a linear function of Pb_air_.

The ADBM shows that the Pb_air _- Pb_blood _varies greatly, for children, according to the age of the subject. It is then necessary to use different functions according both to the age of the children population under exposure and to the duration of the exposure.

Three different scenarios for exposure have then been explored with reference to children. Duration of exposure is set to one year in all the scenarios, because the increment of Pb in the air, Δ-Pb_air_, is the result of an annual Pb emission and because our interest is in the annualized external costs. Three separate age classes have been considered: children aged between 0 and 1 year, between 1 and 2 years, and between 2 and 3 years respectively.

The age-dependent Pb_blood _- Pb_air _functions determining the level of Pb_blood _in children exposed for one year to a constant value of Pb_air _have been calculated with the ADBM as 1.97 μg/dl/μg/m^3 ^for children aged between 0 and 1 year, 2.85 μg/dl/μg/m^3 ^for children aged between 1 and 2 years, and 3.27 μg/dl/μg/m^3 ^for children aged between 2 and 3 years.

Due to different indications in the literature [1,16,17], the external costs have been calculated both considering a damage window of 0<age<2 (which is assumed to be a best-case) and, separately, a damage window of 0<age<3 (worse-case). This should help in assessing how this parameter affects the final results.

### Updated values for the IQ loss - Pb blood function

Pb has a negative impact on children's neurodevelopment. Numerous studies conducted since the late 1980s to date have shown how cognitive impairment is inversely associated with increases in Pb_blood _in children, even at very low Pb_blood _levels (Pb_blood _< 10 μg/dl) [16-19,28-30]. This relationship is measured in IQ lost points for each μg/dl increase in Pb_blood _and is assumed to be linear without threshold [16,31]. Actual levels of children's Pb_blood _are estimated to be between 2-4 μg/dl in Europe [32]. In this paper both actual occurrence of impacts and linearity in the IQ_loss _- Pb_blood _function for marginal increases of Pb_blood _are then assumed. Grosse [33] reports a good summary and discussion of existing values for the IQ_loss _- Pb_blood _function. The author also notices how the function shows a greater slope for lower values of Pb_blood _in children (Pb_blood _< 10 μg/dl) than for higher values (10 μg/dl <Pb_blood _< 20 μg/dl). Following the approach already proposed by Grosse [33], three levels have been selected for the IQ_loss _- Pb_blood _parameter: lower bound, upper bound and base case (Table 1). The IQ_loss_- Pb_blood _slope proposed by Schwartz [16] is derived from a meta-analysis, and has been chosen for the base case. The value for lower bound is to reflect a best case and the upper bound a worst-case scenario. This differentiation should help in assessing how much this parameter affects the results of the analysis.

**Table 1:** IQ - Pb_blood _slope

IQ_loss_- Pb_blood _slope (IQ point/μg/dl)		
**Lower bound**	**Median**	**Upper bound**

0.185	0.257	0.323
[17]	[16]	[16,33]

Values for the IQ_loss _- Pb_blood _slope, with respective referenced source.

### Economic Evaluation of IQ loss

A single pollutant can provoke multiple impacts both with regard to the environment and humans, and not all of them can be expressed in monetary terms. In the specific case of Pb, relevant impacts on human health are those related to anaemia in adults and loss of IQ in children [3]. Monetary measures of IQ loss in children can be calculated according to methodologies developed in the environmental economics literature [1,7,33-35]. The IQ loss is according to Spadaro and Rabl likely to be "...probably the dominant part of the total damage cost of Pb" [1]. The basic principle underlying monetization of this impact is that a loss in cognitive ability occurring in childhood affects future performance in school and work, with a consequent measurable economic loss in lifetime earnings, on a statistical basis. A relationship between IQ loss and percentage reduction in lifetime earnings has been identified by Salkever [35]. Salkever shows that while loss of 1 IQ point causes a loss in lifetime income of 1.9% for men, the loss for women is higher, 3.2%. In the present study the Salkever methodology has been used to derive impacts on lifetime earnings with reference to Danish relative prices and income levels (Jensen J.: Danish life income figures and valuation of IQ, unpublished). The net present value of lifetime income is calculated taking into account gender- and age-specific survival rates for Denmark, while assuming an annual productivity growth of 1% per annum. We use a discount rate of 3%. Lifetime incomes are gender specific and the impact of IQ loss expresses itself differently for men and for women; however, for the final valuation these differences are averaged out. A loss of 1 IQ-point for a Danish citizen is here valued at 18,918 Euros (2006 prices). For sensitivity we also calculated the results with a discount rate of 1.4%, cf. the Stern report [36] and we obtained a value of 37,069 Euros (2006 prices) for each IQ-point loss.

### Calculation of external costs: procedure

The procedure behind IPA can be described with a number of equations. First, the Δ-Pb_air _value for each selected grid cell i (cells where both a value for Δ-Pb_air _is calculated and an exposed population is present) is multiplied by the CRF function that relates Pb_blood _to Pb_air _in children aged between 0 and 1 year, in order to obtain the marginal increase Δ-Pb_blood _(3).(3)

Δ-Pb_blood _is then multiplied by the value of the IQ_loss _- Pb_blood _slope and the marginal loss Δ-IQ_loss _is obtained (4).(4)

Δ-IQ_loss _is multiplied by the cost of one point of IQ_loss _to obtain the marginal monetary loss Δ-€_loss _(5).(5)

A monetary value related to the increase in Pb_air _is now associated to each considered grid cell i. This value needs to be multiplied for the number n_i _of children aged between 0 and 1 years present in the cell. The total cost for each single cell is obtained. This needs to be summarized across all the considered cells. The total cost of the Pb emissions Total_ €_loss _is then obtained (6).(6)

In order to obtain a cost per kg it is necessary to divide the Total_ €_loss _by the total amount of Pb emitted, in kg (7).(7)

The external cost per kg of emitted Pb for the population aged between 0 and 1 year is thereby determined. With the same procedure, the external cost aggregated over the population aged between 1 and 2 years is calculated, using the CRF that relates Pb_blood _to Pb_air _in children aged between 1 and 2 years. The two values for external costs are then added in order to obtain the total external cost of the Pb emission from the source via the inhalation pathway.

## Results

Table 2 reports an example of calculation of External Costs choosing as parameters, respectively: year 2000, base case value for the IQ_loss_- Pb_blood _slope, discount rate of 3%, and damage window for Pb impacts of 3 years from birth (0<age<3). The value of 83 Euros is the cost of each extra kg of Pb emitted, when the population is exposed via the inhalation pathway.

**Table 2:** Calculation of external costs (example)

Parameter	Value			Measure Unit
		
	0< age < 1	1 < age < 2	2 < age < 3	
Pb_blood _- Pb_air_	1.97	2.85	3.27	μg/dl/μg/m^3^
IQ_loss _- Pb_blood_	0.257	0.257	0.257	IQ point/μg/dl
IQ_loss _- Pb_air_	0.506	0.732	0.840	IQ point/μg/m^3^
				
Cost of IQ_loss_	18918	18918	18918	€/IQ point
Pb_emitted_	969	969	969	kg/year
Population	19885	19428	19171	Nr. of children

Aggregated Monetary_loss_	20385	28259	31398	€
Monetary_loss _- Pb_emitted_	21	29	32	€/kg

Total		83		€/kg

Example of values used for year 2000, base scenario, discount rate 3%, damage window for Pb impacts 0 < age < 3.

Values of external costs of Pb emissions, in Euro per kg Pb emitted, for exposure via inhalation and for the different meteorological conditions of the years 2000, 2001 and 2002, are reported in Table 3. All the parameters and data used have been presented in the "Methods" section. Results are in Table 3 organized considering the different possible choices for the key parameters of: IQ_loss_- Pb_blood _slope, discount rate, and Pb impacts damage window. This approach on the variability of parameters and results cannot be considered a complete sensitivity analysis, but represents an indication of the sensitivity and uncertainty involved.

**Table 3:** Pb external costs

**Euro Loss/Pb kg emitted (€/kg)**		**0< age < 2**	**0< age < 3**	**0< age < 2**	**0< age < 3**
**Discount rate**		**3%**		**1.4%**	
		
2000	lower	36	59	71	117
	base	50	83	98	162
	upper	63	104	124	203
					
2001	lower	26	43	51	84
	base	36	59	71	117
	upper	45	75	89	146
					
2002	lower	18	30	35	58
	base	25	41	48	81
	upper	31	52	61	102

Calculated values for External costs, in €/kg, referring to the different meteorological years (2000, 2001, 2002). Various cases are presented: lower-upper bounds and base case regarding the IQ_loss _- Pb_blood _function, discount rates of 3% and 0.1% for the monetary value of 1 point IQ_loss _for Danish conditions, damage window for Pb impacts (0 < age < 2 and 0 < age < 3 years respectively).

The values of 83, 59 and 41 Euro/kg Pb emitted for the meteorological years 2000, 2001, 2002 respectively (Table 3) using a damage window of 0<age<3 as suggested by Schwartz [16] and a discount rate of 3% are here considered as the reference ones, the others can be useful from a sensitivity perspective. Results can be discussed considering the characteristics of the key parameters in the method.

## Discussion

Consideration can be given to the results obtained with regard to their robustness, the degree to which they are affected by the different parameters, how similar they are to those from previous studies, but also of weak, strong and novel aspects of the method adopted.

There are likely to be some differences in the "behaviour" of different Pb species, but we are not able to account in detail for these. We apply a "toxicological" approach (cf. replies to Duffus editorial [37]) based on "Total Pb": a pooled value accounting for all the relevant Pb species. In relation the IPA steps the following qualifications should be observed;

-) Emission and dispersion: the measurements determine only "Total Pb" in flue gases. Pb is adsorbed on PM and the dispersion model does not account for differences in the dispersion between various species of Pb.

-) Exposure: while the breathing rate is independent from the Pb species, the absorption rate is based only on the bio-available fraction of Pb. Not all the inhaled Pb is assumed to be absorbed. In this way a pooled estimate of the relevant Pb species appears.

-) Bio-Accumulation: the ADBM parameters have been calculated from bio-monitoring data and consider "Total Pb" in the different organs/tissues as a pooled figure for the relevant Pb species.

-) IQ loss - Pb_blood _function: is in the literature calculated from bio monitoring data reporting the total (pooled) Pb content in blood.

The topic of IQ loss from Pb exposure in childhood remains of concern, perhaps in particular in developing countries. With regard to the IQ_loss _- Pb_blood _function, several studies [8,16,18,19,28,29,31] address the topic; most recently Solon [30], with a site study in the Philippines, identifying a relatively high value for the relationship between Pb and IQ (A 3.32 point decline in IQ for each μg/dl increase in Pb_blood _in children aged 6 months to 3 years). The meta-review of Schwartz [16], however, is widely considered to represent the best global estimate at the present time [1,33]. Schwartz refers to the US population which eases the application of his features to European conditions, considering that both contexts deal with populations in developed countries. The choice of IQ_loss _- Pb_blood _function affects the final results only to some extent; the selection of damage window contributes more strongly, evidently because definition of the number of children exposed is involved. By using the ADBM we can give a good definition of the Pb_blood _- Pb_air _function for different ages and times of chronic exposure, but then we need to select the exposed population carefully. The state of the art of current research does not give precise indications of the damage window for Pb-related neurodevelopmental impacts. The above cited studies regarding the loss of IQ related to Pb in blood have been performed measuring IQ value (or equivalent indices for intelligence) at the minimum age of 6 months, and focusing mainly on the influence of covariates such as gender, sex, and level of Pb_blood_, without trying to identify a specific moment when impact is supposed to occur. In general, a valid assumption, considering that the process of neurodevelopment in children is higher in the first 2-3 years immediately after birth, is that the impacts of potential impairment due to Pb should be greater in these early years. The scenarios considering 0 < age <2 and 0 < age < 3 years as the damage window are included to demonstrate the relative significance of the age parameter choice. In comparison the different spatial dispersion of the contaminant, pending only on meteorology and resulting in a different amount of population under exposure, displays some significance too.

It is interesting to compare the results obtained in the present study with findings from earlier studies. As the main references for comparison we can take the work performed in the Espreme project [3] and the work of Spadaro [1], respectively. Results proposed in the present study are of the same order of magnitude as those presented in the Espreme project with regard to the impacts of Pb via inhalation. Espreme reports a value of 24.97 Euro/kg for Denmark; relatively close to the values here presented. However, there are considerable differences in the two approaches: in the Espreme project the spatial scale is different, the atmospheric dispersion modelling is performed at the European level and values of external costs are calculated on a national not a local level. Furthermore, there is no use of any biokinetic model: impacts of Pb on IQ are calculated using an exposure period of 5 years and a population of children under exposure corresponding to the 20% of the total population. Finally, the evaluation method is not clearly specified (We assume the loss in lifetime earnings is used). The approach used by Spadaro is different again. Spadaro considers a Pb_blood_- Pb_air _function of 5 μg/dl/μg/m^3 ^for incremental exposure to Pb in ambient air, citing a reference from a UK study. The number of children under exposure is assumed to be the 1.1% of the total population, regardless of any damage window for Pb impact. The monetization is determined using the relationship between loss in IQ and decrease in income. The values for external costs reported by Spadaro are two orders of magnitude higher than the values here reported for Denmark. They are reported at an EU level and they are not limited to inhalation but include also ingestion of Pb, because the measured Pb_blood- _Pb_air_function implicitly accounts for the ingestion pathway also. The difference between the inhalation and the ingestion pathway is significant. If inhalation is assumed to account only for 4% of the total intake of Pb belonging from the source, as has been suggested above, then it is reasonable to expect a value for the external costs regarding the total Pb intake (inhalation + ingestion) that is somewhere between one and two orders of magnitude higher than when referring to inhalation only.

In our study we chose to focus on the inhalation pathway, because the link between air emission and inhalation is direct and no multimedia distribution is involved. It is also difficult to distinguish the contribution of different exposure pathways to the total Pb exposure and to the increase in Pb_blood_, and in particular to relate such increment to the specific considered source of emission. The ADBM has been here used in order to isolate and specify the relationship existing between Pb_air _and Pb_blood _and separate this from other intake pathways, so the model allows for a better quantification of this source and this exposure pathway contribution to the overall impact. However, the total impact is likely to be much greater. Under natural background exposure conditions the ingestion of Pb is, in terms of Pb intake (μg/day), around two orders of magnitude greater than the inhalation [1]. This does not anyway necessarily imply that ingestion of Pb emitted from the source will be also two orders of magnitude greater the inhalation. The link between the source and the intake (and then impact) trough the ingestion pathway is uncertain: deposition and redistribution in the soil could dilute the contaminant concentration so much that ingestion of Pb emitted from the source could not be significant. It could be possible to find a relationship between Pb_soil _- Pb_blood _by using the ADBM and applying the same approach that has been explained above for inhalation. Ideally this could allow for accounting of the incremental impact relating to the ingestion of Pb emitted from the source and consecutively deposited in the soil. The problem is that, at the moment, the uncertainties in the establishment of a multimedia link between source and impact via ingestion, considering the marginality of the source, appear to be quite high. We provide only a partial result, but the methodology based on ADBM piloted here would allow for exploring also the ingestion pathway and so arrive at more robust figures for the external costs of Pb exposure. A multimedia approach will be required as an extension of the modelling framework presented here.

## Conclusions

External costs for marginal Pb emission originating from a waste-to-energy plant have been assessed according to the impact pathway approach and extended with a detailed atmospheric dispersion model and a biokinetic model. The assessment of exposure to Pb for the most sensitive segment of the population has been determined carefully using spatially defined data as well as reference values for Pb intake and Pb impacts. The influence of some key parameters such as the IQ_loss _- Pb_blood _function, the damage window for Pb impacts, and the discount rate has been discussed in a critical perspective. This approach considers only the initial effects via inhalation, and consequently accounts for only a part of the total costs of Pb emissions. Even these conservative values suggest that policies to reduce Pb exposure deserve careful considerations in the policy making process. The importance of assessing the contribution of the ingestion pathway, even if under conditions of greater uncertainty, has been stressed and will be the topic of further research.

## List of Abbreviations

ADBM: Age-Dependent Biokinetic Model; AF: Absorption Factor; CBA: Cost Benefit Analysis; CPR: Personal Registration Code; CRF: Concentration Response Function; ICRP: International Commission for Radiological Protection; IPA: Impact Pathway Approach; IQ: Intelligence Quotient; IR: Intake (Breathing) Rate; LCA: Life Cycle Assessment; NERI: National Environmental Research Institute; OML: Operational Meteorological air-quality Model; PM: Particulate Matter; US- EPA: United States Environmental Protection Agency; WtE: Waste-to-Energy plants.

## Competing interests

The authors declare that they have no competing interests.

## Authors' contributions

MP carried out the determination of population under exposure, age-dependent CRF using the ADBM model and calculation of external costs, and drafted the manuscript. MSA and MT participated in the design and coordination of the study and supervised the work, then contributed to the drafting and revising of the manuscript. LMF carried out the air dispersion modelling and provided the relative data. All authors read and approved the final manuscript.
